# Single‐Dose Pharmacokinetic Assessment of TNX‐102 SL (Cyclobenzaprine HCl Sublingual Tablets): Results From Randomized, Open‐Label Studies in Healthy Volunteers

**DOI:** 10.1002/cpdd.70034

**Published:** 2026-02-26

**Authors:** Bruce L Daugherty, Bernd Meibohm, Gregory M Sullivan, Errol M Gould, Seth Lederman

**Affiliations:** ^1^ Tonix Pharmaceuticals Inc. Berkeley Heights NJ USA; ^2^ University of Tennessee Health Science Center Memphis TN USA; ^3^ Tonix Medicines, Inc Berkeley Heights NJ USA

**Keywords:** cyclobenzaprine, fibromyalgia, sublingual

## Abstract

Daily oral cyclobenzaprine hydrochloride (HCl) has provided transient benefits in fibromyalgia, a chronic pain condition. To improve this effect, we evaluated sublingual formulations designed to drive transmucosal absorption. Two open‐label studies evaluated the pharmacokinetics (PK), tolerability, and relative bioavailability of sublingual cyclobenzaprine HCl in healthy adults. In Study 1 (n = 24), three 2.8 mg sublingual formulations of cyclobenzaprine HCl containing potassium phosphate dibasic (A), sodium phosphate dibasic (B), or trisodium citrate (C) were compared to immediate release (IR) cyclobenzaprine HCl 5 mg. All sublingual formulations showed increased bioavailability (154% [A], 126% [B], and 125% [C]) and rapid absorption. Formulation A demonstrated the most favorable PK, with a ∼3 min absorption lag versus ∼37 min for oral IR, and a 783% higher dose‐normalized AUC_0‐1_. Formulation A was designated TNX‐102 SL for further development. In Study 2 (n = 16), TNX‐102 SL 2.8 and 5.6 mg exhibited dose proportionality and no food effect. Furthermore, this is the first report describing the active metabolite norcyclobenzaprine in clinical studies, showing an elimination half‐life of ∼60 h. Oral hypoesthesia and abnormal taste were the most common adverse events. These findings support TNX‐102 SL as a rapidly absorbed and efficient sublingual tablet formulation of cyclobenzaprine HCl, providing effective transmucosal delivery.

The prevalence of fibromyalgia (FM) in the United States is estimated to be ∼6.4% of the adult general population.^1^ FM is a common chronic pain syndrome characterized by widespread pain, nonrestorative sleep, and fatigue.^2^ Since 2009, no new pharmacotherapies had been approved by the US Food and Drug Administration for FM until the approval of cyclobenzaprine HCl sublingual tablets in 2025.^3^ Before this approval, available treatments had modest efficacy for management of pain associated with FM; did not consistently improve all three symptoms in the FM core triad of pain, nonrestorative sleep, and fatigue; and had suboptimal tolerability that affects persistency of treatment.^4^, ^5^, ^6^

Oral, immediate‐release (IR) cyclobenzaprine HCl (10 mg three times a day) has been approved for short‐term use (2‐3 weeks) in the United States since 1977 as an adjunct to rest and physical therapy for the relief of muscle spasm associated with acute, painful musculoskeletal conditions.^7^ A randomized, double‐blind, placebo‐controlled trial showed that daily bedtime oral cyclobenzaprine HCl treatment was associated with nominal pain improvements compared with placebo after 1 month of treatment but not after 3 or 6 months.^8^ Because FM is a chronic pain condition, the development of oral cyclobenzaprine HCl for treating FM was not pursued due to the lack of sustained benefits. Long‐term treatment with oral cyclobenzaprine HCl is also limited owing to tolerability concerns, including dry mouth, somnolence, dizziness, drowsiness/fatigue, and weight gain.^8^, ^9^ In an 8‐week study,^10^ oral cyclobenzaprine HCl at bedtime was evaluated using lower doses of cyclobenzaprine. In this dose‐escalation study, doses of cyclobenzaprine HCl below 5 mg at bedtime were shown to be active in targeting nonrestorative sleep and were correlated with improvement in several daytime symptoms.^10^

Cyclobenzaprine, a tertiary amine tricyclic, is metabolized to its desmethyl derivative, norcyclobenzaprine, a secondary amine tricyclic, via hepatic CYP3A4 and CYP1A1/2, and to a lesser extent, CYP2D6.^11^, ^12^, ^13^ Norcyclobenzaprine is a persistent active major metabolite. Moreover, norcyclobenzaprine HCl may limit the durability of oral cyclobenzaprine's activity in treating FM as a bedtime medicine because norcyclobenzaprine has a several‐day half‐life that leads to accumulation to a steady state over several weeks, and it is a uniquely potent inhibitor of the norepinephrine transporter, which is expected to impair sleep quality.^14^ Therefore, we reasoned that bypassing first‐pass hepatic metabolism via transmucosal absorption may result in lower exposure to norcyclobenzaprine compared with oral cyclobenzaprine HCl IR, and lower exposure to norcyclobenzaprine may improve the durability of efficacy of chronic bedtime therapy for FM.^7^ In this scenario, reduced norcyclobenzaprine accumulation may confer durability to the otherwise transient effect of oral cyclobenzaprine on pain in FM.

Based on these findings, a sublingual formulation of cyclobenzaprine and potassium phosphate dibasic (K_2_HPO_4_), designated TNX‐102 SL, was developed. In addition to K_2_HPO_4_, TNX‐102 SL contains a eutectic of cyclobenzaprine HCl and mannitol, which protects cyclobenzaprine from basifying agents (unpublished observations). TNX‐102 SL is a small, rapidly disintegrating tablet containing 2.8 mg of cyclobenzaprine HCl in a eutectic formulation for sublingual administration and transmucosal absorption. In two Phase 3 multicenter randomized, double‐blind, placebo‐controlled studies in patients with FM, treatment with sublingual cyclobenzaprine HCl 5.6 mg (two tablets of 2.8 mg of cyclobenzaprine HCl) at bedtime resulted in significant reductions in the prespecified primary endpoint of daily pain intensity and was generally well tolerated.^15^, ^16^

Here, we report the results of two Phase 1 studies in healthy adults: (1) comparing the pharmacokinetics and tolerability of three different formulations of sublingual cyclobenzaprine HCl, each distinguished by a specific basifying agent, to oral cyclobenzaprine HCl 5 mg IR with the goal of selecting a lead sublingual formulation for further clinical development; and (2) a follow‐up study evaluating the dose‐proportionality and food effect of the selected sublingual formulation, TNX‐102 SL. TNX‐102 SL was FDA approved in August 2025 for the bedtime treatment of FM.^3^

## Methods

### Study 1

#### Study Design

Study 1 was a single‐center, randomized, single‐dose, open‐label, comparative, parallel‐design, pharmacokinetics and safety study (NCT01889173; registered 6/28/2013) performed under fasting conditions in healthy adults, conducted at Syneos Health Clinique in Québec City, Canada. Each participant who qualified for the study treatment was sequentially assigned a subject number prior to receiving any study treatment. The subject numbers corresponded to the randomization scheme, and the randomization schedule was generated by inVentiv. Eligible participants were admitted to the study unit the day before dose administration and the morning after admission were randomly assigned to and received one of four treatments: sublingual cyclobenzaprine HCl 2.8 mg with K_2_HPO_4_ (Formulation A), sublingual cyclobenzaprine HCl 2.8 mg with sodium phosphate dibasic (Na_2_HPO_4_) (Formulation B), sublingual cyclobenzaprine HCl 2.8 mg with trisodium citrate (Na_3_C_6_H_5_O_7_) (Formulation C), or oral cyclobenzaprine HCl 5 mg IR. Participants remained at the study site from ≥10 h before dosing until the 48‐h post‐dose blood sample was collected.

The 2.8 mg TNX‐102 SL tablet dose in Study 1 was selected based on an 8‐week, Phase 2a dose‐escalation study of oral cyclobenzaprine.^10^ Patients started with a 1‐mg dose, which could be increased each week by 1 mg for a maximal dose of 4 mg, if clinically indicated and based on tolerability. At the end of 8 weeks, the mean average daily dose of cyclobenzaprine capsules was 3.1 mg.^10^ Due to the increased bioavailability of plasma cyclobenzaprine for the sublingual formulation, the sublingual dose of cyclobenzaprine (2.8 mg) was estimated to yield approximately equivalent peak exposure (C_max_) to a 3.1‐mg oral dose, a dose shown to be associated with positive clinical effects in the Phase 2a study.^10^ Based on pharmacokinetic (PK) studies in healthy adult volunteers, a dose of 2.8 mg was expected to be bioequivalent to an oral capsule dose based on systemic exposure. The dose of the three investigational TNX‐102 SL tablet formulations evaluated in this study (2.8 mg) was lower than the lowest commercially available dose of oral cyclobenzaprine (5 mg) and was therefore not expected to pose any increased safety risk to the volunteers.

The protocol, protocol amendment, informed consent form, and relevant associated documents were reviewed by an institutional review board (IRB; Institutional Review Board Services) before screening and enrollment. Participants provided written informed consent in accordance with local regulations before conducting any study‐specific procedures. All study procedures were conducted in compliance with Good Clinical Practice according to the International Council for Harmonisation guidelines.

#### Participants

Eligible participants were healthy adults 18‐65 years of age (inclusive) with a body mass index (BMI) >18.5 to <30.0 kg/m^2^ who did not use tobacco‐ or nicotine‐containing products within 3 months of screening and who were healthy as defined by no clinically significant disease or surgery within the preceding 8 weeks or history of clinically significant disease. Key exclusion criteria were the use of a potent cytochrome P450 (CYP) 3A4 inhibitor, monoamine oxidase inhibitor antidepressants, or any drug known to inhibit hepatic drug metabolism within 30 days prior to dosing; any clinically significant electrocardiogram (ECG) abnormalities; any significant abnormality or clinically significant abnormal laboratory test result found during medical screening; positive urine drug test or alcohol breath test at screening or time of admission; use of any supplement or food known to induce or inhibit drug metabolism within 14 days prior to dosing; use of cyclobenzaprine HCl within 60 days prior to dosing; positive pregnancy test, breastfeeding, or lactation; and the presence of dentures, piercing, or braces that might interfere with oral cavity examination upon and after sublingual dosing.

#### Pharmacokinetic Sampling and Analysis

Blood samples were taken for PK analysis from all participants 30 min before dosing; 2, 3.5, 5, 10, 20, 30, and 45 min after dosing; and 1, 2, 2.5, 3, 3.33, 3.67, 4, 4.33, 4.67, 5, 5.5, 6, 8, 12, 16, 24, 36, and 48 h after dosing. The total volume of blood collected for all study assessments did not exceed 266 mL for the entire study. All blood samples were taken into EDTA collection tubes, cooled in an ice bath, and centrifuged at approximately 1900 × *g* for ≥10 min at approximately 4°C. Two aliquots of ≥0.5 mL of plasma were dispensed into polypropylene tubes, transferred to a −80°C freezer, and then transferred to the bioanalytical facility.

A single urine sample was collected within 30 min pre‐dose, and intra‐individual samples were pooled for the duration of the dosing period from 0 to 24 and 24 to 48 h post‐dose. All urine samples were collected at room temperature in glass containers, pooled into 5‐L glass bottles, and stored at 4°C until the end of the sampling period. Approximately 10 mL of sample was divided into four aliquots of equal volume (≥2.0 mL) and transferred to borosilicate screw cap culture tubes.

Cyclobenzaprine concentrations in plasma were assessed using high‐performance liquid chromatography with tandem mass spectrometry (HPLC‐MS/MS). Cyclobenzaprine and the internal standard (IS) cyclobenzaprine‐d3 were extracted from plasma (0.200 mL aliquots) using an automated liquid–liquid extraction with methyl‐*tert*‐butyl ether. The extracted samples were eluted on a gradient and analyzed using an MS/MS detector with an API 5000 (Sciex; Toronto, Canada). Quantification was based on the peak area ratio of the analytes versus the stable labeled IS, with a weighted (1/×2) linear regression to determine the concentration of the analytes. The validated assay range was 50‐10,000 pg/mL for cyclobenzaprine. All plasma concentration values and urine concentration values below the lower limit of quantitation were replaced by “0,” as were PK parameter calculations for samples with no reportable value occurring before dosing. All samples with no reportable value observed after drug administration were treated as missing values.

Plasma concentration values of cyclobenzaprine were used to calculate the following parameters using standard noncompartmental methods: area under the concentration‐versus‐time curve (AUC) from time zero to the last measurable concentration (AUC_0‐t_), calculated using the linear trapezoidal method; AUC from time zero to infinity (AUC_0‐∞_, extrapolated); residual area, calculated as 100 × (1 – AUC_0‐t_/AUC_0‐∞_); maximum measured plasma concentration (C_max_); time to C_max_ (T_max_); and elimination half‐life (t_1/2_). Relative bioavailability (F_rel_) was calculated for the sublingual formulations versus the oral formulation of cyclobenzaprine HCl using the mean AUC_0‐∞_ and correcting for dose differences using the formula, F_rel_ = 100 × [dose (oral) × AUC (sublingual)]/[dose (sublingual) × AUC (oral)]. In an exploratory analysis, partial AUC values for cyclobenzaprine in plasma were calculated and summarized using descriptive statistics for all four treatment groups over the following intervals: 0‐1, 0‐2, 0‐8, 8‐24, and 0‐24 h. Plasma concentration‐over‐time profiles 0‐2 h post‐dose were compared for Formulations A, B, and C versus oral IR cyclobenzaprine HCl.

Cumulative urinary excretion of cyclobenzaprine, norcyclobenzaprine, and cyclobenzaprine‐N‐glucuronide from time 0 to time t (Ae_0‐t_) was calculated as the sum of the amounts excreted over each collection interval; the amount excreted in urine for each time interval was calculated as the urine concentration multiplied by the urine volume. The maximum rate of urinary excretion was calculated by dividing the amount of drug excreted in each collection interval by the time over which it was collected.

#### Model‐Based Analysis of Cyclobenzaprine Pharmacokinetic Data

Cyclobenzaprine plasma concentration data were obtained from six participants in each treatment group. A model‐based PK analysis was performed by nonlinear regression. A two‐compartment mammillary model with a first‐order absorption and absorption lag time was used. These parameters were defined in relation to clearance and volume. Plasma concentrations below the specified lower limit of quantification (50 pg/mL) were recorded as 0 for the analysis. Due to the short sampling window (48 h) compared to the terminal half‐life of cyclobenzaprine (30‐35 h), analysis of individual concentration–time profiles failed for many participants. However, average concentration–time profiles were calculated for each treatment group and then modeled.

#### Safety Assessments

Safety was assessed at scheduled times while the participant was at the study site and included adverse event (AE) reporting, clinical laboratory parameters, vital signs, and ECG. A follow‐up telephone call 10 ± 3 days after study drug administration was conducted to solicit information on AEs. Any clinically significant abnormal laboratory results or other abnormalities occurring during the course of the study were followed until resolution or until the investigator determined the values were not likely to return to normal.

#### Statistical Analysis

A sample size of approximately 24 participants, with 6 participants randomly assigned to each of four single‐dose formulations/treatment, was considered sufficient to meet the study objectives. The PK population included all participants who completed the study without major protocol violation(s) and whose PK profile was adequately characterized. Participants were excluded from the PK population if there was clear evidence of protocol noncompliance or if the pre‐dose plasma cyclobenzaprine HCl concentration was >5% of the C_max_ value. The safety population included all participants who received the study medication.

Individual and mean concentration‐versus‐time curves were determined using linear and semi‐log scales. Pharmacokinetic parameters were tabulated for each treatment per analyte and summarized using descriptive statistics. For all analytes, analysis of variance (ANOVA) was performed on untransformed T_max_, K_el_, and t_1/2_ and on ln‐transformed AUC_0‐t_, AUC_0‐∞_, and C_max_ using a general linear model (GLM) at the 0.05 α level. The ratio of least squares (LS) means (Formulations A/D, B/D, and C/D) and 90% CIs were calculated for AUC_0‐t_, AUC_0‐∞_, and C_max_.

Urine concentrations and urinary excretion of cyclobenzaprine, norcyclobenzaprine, and cyclobenzaprine‐N‐glucuronide were evaluated and summarized by treatment using descriptive statistics. The ratios of LS means (Formulations A/D, B/D, and C/D) were calculated for ln‐transformed Ae_0‐t_ and R_max_ using GLM procedures.

### Study 2

#### Study Design

Study 2 was a single‐center, randomized, single‐dose, open‐label, three‐period (NCT04164719; registered 11/13/2019), crossover study in healthy adults designed to assess the selected formulation of sublingual cyclobenzaprine HCl from Study 1 (Formulation A, TNX‐102 SL, formulated with K_2_HPO_4_). The study was conducted at Syneos Health Clinique in Québec, Canada. Eligible participants were admitted to the study unit ≥10 h before study drug administration and remained until 24 h post‐dose. Participants were randomized using a randomization schedule generated by Syneos Health with a validated proprietary computer software program (Statistical Analysis System, Version 9.2) and reviewed by a biostatistician. Participants who met the eligibility criteria were randomly assigned to receive Formulation A, B, or C according to the three‐period, six‐sequence, block randomization scheme. During each period, participants received one of three treatments and conditions (randomized upon arrival at the study unit): sublingual TNX‐102 SL 2.8 mg under fasting conditions, sublingual TNX‐102 SL 5.6 mg (2 × 2.8 mg tablets) under fasting conditions, or sublingual TNX‐102 SL 5.6 mg (2 × 2.8 mg tablets) under fed conditions. For the fed condition, participants received a high‐fat, high‐calorie breakfast 30 min before study drug administration and were required to finish the meal within the 30‐min period. After leaving the study site, participants returned for subsequent blood draws. Each treatment period was separated by a 28‐day washout period.

In Study 2, a dose of 5.6 mg of TNX‐102 SL was chosen. Cyclobenzaprine is commonly used at 5‐10 mg three times daily to treat muscle spasms and is generally well tolerated at these doses. In a previous Phase 3 FM study, treatment with TNX‐102 SL 2.8 mg showed clinically meaningful efficacy across most secondary and sensitivity analyses, and the study's primary efficacy endpoint (30% responder analysis) showed a positive trend (*P* = 0.095) but was not statistically significant.^15^ Based on these results, we expected that a higher dose of TNX‐102 SL (i.e., 5.6 mg) would have the potential to establish substantial evidence of efficacy and maintain an acceptable safety and tolerability profile in patients with FM. Overall, the TNX‐102 SL 5.6‐mg dose was substantially below the recommended dose range for currently marketed cyclobenzaprine products, AMRIX and FLEXERIL; however, it is reasonable to expect that better tolerability may be achieved at these lower doses when administered sublingually at bedtime, when drowsiness would be a positive attribute for targeting the sleep disturbance of FM, rather than a negative effect.

The protocol, protocol amendment, informed consent form, and relevant associated documents were reviewed by an IRB (Advarra) before screening and enrollment. Participants provided written informed consent in accordance with local regulations before conducting any study‐specific procedures. All study procedures were conducted in compliance with Good Clinical Practice according to the International Council for Harmonisation guidelines.

#### Participants

Eligible participants were healthy adults 18‐65 years of age (inclusive) with a BMI >18.5 to <30.0 kg/m^2^ and body weight ≥50 kg for males and ≥45 kg for females. Participants could not use or have used tobacco or nicotine products within 3 months prior to screening and were healthy as defined by no history of clinically significant disease and no clinically significant illness or surgery within the preceding 8 weeks. Key exclusion criteria followed those of Study 1.

#### Pharmacokinetic Sampling and Analysis

Blood samples were taken for PK analysis from all participants at 0.5, 1, 2, 3, 4, 4.33, 4.67, 5, 5.33, 5.67, 6, 8, 10, 12, 13, 14, 15, 16, 18, 20, 22, 24, 36, 48, 72, 96, 120, 144, 168, 216, 264, 312, and 360 h post‐dose. Blood sample collection and processing and PK parameters and analysis followed that of Study 1.

#### Safety Assessments

Safety was assessed at scheduled times while the participant was at the study site and included AE reporting, clinical laboratory parameters, vital signs, and ECG. A follow‐up telephone call 10 ± 3 days after study drug administration was conducted to solicit information on AEs. Any clinically significant abnormal laboratory results or other abnormalities occurring during the course of the study were followed until resolution or until the investigator determined the values were not likely to return to normal.

#### Statistical Analysis

A total of 16 healthy adult male or female participants were dosed. Efforts were made to enroll a balanced number of male and female participants. The PK population included all participants who completed ≥2 periods and whose PK profile was adequately characterized. Participants were excluded from the PK population if there was clear evidence of protocol noncompliance or if the pre‐dose plasma cyclobenzaprine HCl concentration was >5% of the C_max_ value. The safety population included all participants who received ≥1 dose of study medication.

Individual and mean concentration‐versus‐time curves were determined using linear and semi‐log scales. Pharmacokinetic parameters were summarized using descriptive statistics. Dose proportionality and food effect were assessed using an ANOVA on untransformed T_max_, K_el_, and t_1/2_ and on ln‐transformed AUC_0‐t_, AUC_0‐∞_, and C_max_ using a GLM at the 0.05 α level. The ratio of geometric means (2.8‐5.6 mg) and 90% CIs were calculated for dose‐normalized AUC_0‐t_, AUC_0‐∞_, and C_max_. Dose proportionality and lack of food effect were concluded if the 90% CI for the ratio of geometric means was between 80% and 125%.

## Results

### Study 1

#### Participants

Between June 4, 2013, and July 2, 2013, a total of 27 participants were enrolled, and 24 were randomly assigned to the four treatment groups. No notable differences were observed among the four treatment groups with respect to demographic and baseline characteristics (Table 1). The mean participant age was 36.2 years and 58% were female.

**Table 1:** Study 1: Demographic and Baseline Characteristics (Safety Population)

	Formulation A:	Formulation B:	Formulation C:	
Characteristic	Sublingual Cyclobenzaprine HCl 2.8 mg (Potassium Phosphate Dibasic) (n = 6)	Sublingual Cyclobenzaprine HCl 2.8 mg (Sodium Phosphate Dibasic) (n = 6)	Sublingual Cyclobenzaprine HCl 2.8 mg (Trisodium Citrate) (n = 6)	Oral Cyclobenzaprine HCl 5 mg IR (n = 6)
Mean age (range), years	36.7 (19‐56)	33.3 (24‐56)	37.5 (21‐49)	37.3 (22‐59)
Sex, n (%)				
Female	4 (67)	3 (50)	4 (67)	3 (50)
Male	2 (33)	3 (50)	2 (33)	3 (50)
Race, n (%)				
White	5 (83)	6 (100)	6 (100)	6 (100)
Asian	1 (17)	0	0	0
Ethnicity, n (%)				
Not Hispanic	5 (83)	5 (83)	6 (100)	5 (83)
Hispanic	1 (17)	1 (17)	0	1 (17)
Mean BMI (range), kg/m^2^	26.2 (21.2‐29.0)	23.8 (21.6‐27.3)	25.9 (21.1‐29.5)	25.9 (22.5‐28.3)

BMI, body mass index; IR, immediate release.

#### Cyclobenzaprine Pharmacokinetics in Plasma

**Figure 1:**
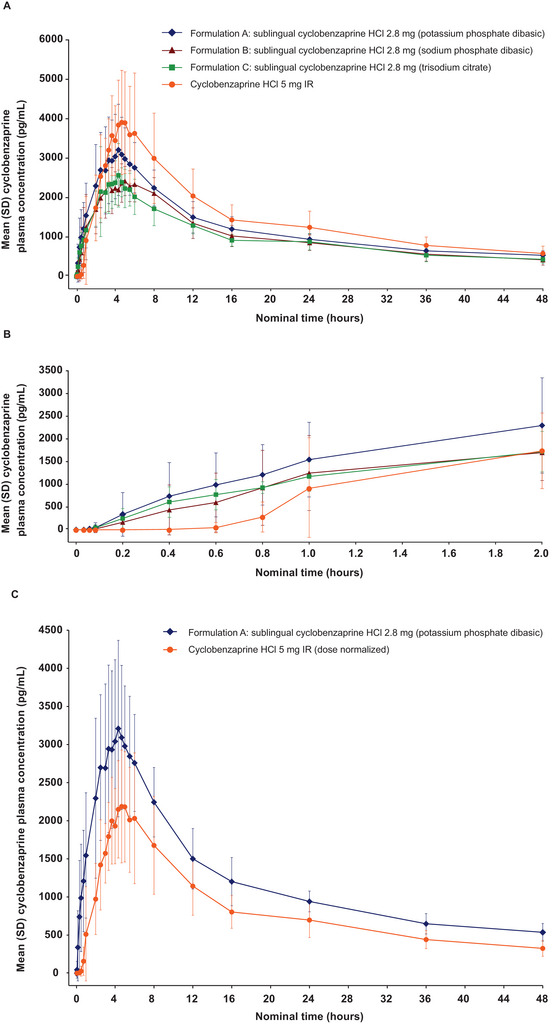
Study 1: Plasma concentration of cyclobenzaprine (A) over time; (B) from 0 to 2 h post‐dose for the 3 formulations of sublingual cyclobenzaprine HCl and oral cyclobenzaprine HCl 5 mg IR (pharmacokinetic population); and (C) with dose normalized for oral cyclobenzaprine HCl 5 mg IR. IR, immediate release.

Mean calculated PK parameters for plasma cyclobenzaprine following one of the three sublingual cyclobenzaprine HCl 2.8 mg formulations or oral IR cyclobenzaprine HCl 5 mg IR are summarized in Table 2. Mean plasma cyclobenzaprine concentration over 48 h after a single dose of study medication was highest with oral IR cyclobenzaprine HCl compared with the three formulations of sublingual cyclobenzaprine HCl (Figure 1). LS mean ratios of C_max_ and AUC_0‐∞_ for Formulation A were 81% and 87%, respectively, those of oral IR cyclobenzaprine HCl. C_max_ for Formulations B and C was lower than for Formulation A and was 66% and 68%, respectively, that of oral IR cyclobenzaprine HCl. Similarly, AUC_0‐∞_ was 71% and 71%, respectively, versus oral IR cyclobenzaprine HCl. The mean F_rel_ of Formulations A, B, and C versus oral IR cyclobenzaprine HCl were 154%, 126%, and 125%, respectively. Cyclobenzaprine T_max_ occurred between 4.2 and 4.7 h for Formulations A, B, and C versus 4.0 h for oral IR cyclobenzaprine HCl, and the mean t_1/2_ was 24.3‐27.4 h after dosing for all treatments (A–C and oral IR cyclobenzaprine HCl). There were no statistically significant differences between treatments for T_max_, K_el_, and t_1/2_.

**Table 2:** Study 1: Single‐Dose Pharmacokinetic Parameters for Cyclobenzaprine in Plasma (Pharmacokinetic Population)

	Formulation A:	Formulation B:	Formulation C:	
Parameter	Sublingual Cyclobenzaprine HCl 2.8 mg (Potassium Phosphate Dibasic) (n = 6)	Sublingual Cyclobenzaprine HCl 2.8 mg (Sodium Phosphate Dibasic) (n = 6)	Sublingual Cyclobenzaprine HCl 2.8 mg (Trisodium Citrate) (n = 6)	Oral Cyclobenzaprine HCl 5 mg IR (n = 6)
Cyclobenzaprine				
AUC_0‐t_, mean (SD), ng·h/mL	57.4 (10.7)	49.0 (10.4)	46.8 (9.2)	69.5 (18.8)
Ratio (90% geometric CI), %^a^	83.5 (67.3‐103.6)	71.2 (57.4‐88.3)	68.1 (54.9‐84.5)	‐
AUC_0‐∞_, mean (SD), ng·h/mL	78.8 (15.9)	64.4 (15.6)	64.1 (14.5)	91.4 (24.2)
Ratio (90% geometric CI), %^a^	87.2 (68.4‐111.1)	70.6 (55.4‐90.1)	70.8 (55.5‐90.3)	‐
Residual area, %	26.9 (3.4)	23.0 (6.7)	26.6 (3.5)	23.2 (10.2)
C_max_, mean (SD), ng/mL	3.4 (1.0)	2.7 (0.5)	2.9 (0.9)	4.3 (1.4)
Ratio (90% geometric CI), %^a^	80.6 (61.0‐106.6)	66.4 (50.3‐87.8)	68.1 (51.5‐90.0)	‐
T_max_, median (range), h	4.3 (2.0, 6.0)	4.7 (3.3, 6.0)	4.2 (2.5, 4.7)	4.0 (3.3, 6.0)
K_el_, mean, L/h	0.026 (0.003)	0.029 (0.005)	0.026 (0.004)	0.030 (0.008)
t_1/2_, mean (SD), h	27.4 (3.3)	24.3 (4.6)	27.1 (3.4)	25.1 (9.2)
F_rel_, %^b^	153.9	125.8	125.3	‐

AUC, area under the plasma concentration‐versus‐time curve; AUC_0‐t_, AUC from time 0 to last measurable concentration; AUC_0‐∞_, AUC from time 0 extrapolated to infinity; C_max_, maximum measured plasma concentration; F_rel_, relative bioavailability; IR, immediate release; K_el_, elimination constant; t_1/2_, elimination half‐life; T_max_, time of the maximum measured plasma concentration.

a Normalized to oral IR cyclobenzaprine HCl. Ratios were calculated using LS means; 90% geometric CIs were used for the ln‐transformed data.

b Calculated using the dose‐normalized mean values for each treatment, not for each participant, in relation to oral IR cyclobenzaprine HCl.

Compared with oral IR cyclobenzaprine HCl, the mean AUC_0‐1_ of cyclobenzaprine in plasma with Formulation A was approximately 4.4‐fold higher (0.88 vs 0.20 ng·h/mL, respectively), and AUC_0‐2_ was almost 2‐fold higher (2.8 vs 1.5 ng·h/mL) (Table S1). When doses were normalized, the mean ratio of Formulation A to oral IR cyclobenzaprine HCl was higher for AUC_0‐1_ (783%) and AUC_0‐2_ (327%) of cyclobenzaprine in the plasma (Table S1). Although several parameters cannot be reliably estimated from the data, likely due to the limited sampling time period, the estimates for the absorption lag time were precise. In Formulations A, B, and C, the absorption lag time was substantially shorter (∼3 min), with approximately a 12‐fold difference compared with oral IR cyclobenzaprine HCl (∼37 min). Mean AUC_8‐24_ for cyclobenzaprine was approximately 22% lower for Formulation A versus oral IR cyclobenzaprine HCl. When doses are normalized, the ratio of Formulation A to oral IR cyclobenzaprine HCl was 139% for AUC_8‐24_. A similar pattern with respect to a faster rate of cyclobenzaprine absorption during the first 2 h after sublingual cyclobenzaprine HCl administration was also seen for Formulations B and C compared with oral IR cyclobenzaprine HCl.

#### Norcyclobenzaprine Pharmacokinetics in Plasma

Although PK data for norcyclobenzaprine were collected, they are not reported in this study. Reliable estimation of PK parameters was not possible due to the small sample size and limited sampling duration (48 h), which was insufficient relative to the longer half‐life of norcyclobenzaprine (see Study 2 results in Table 5).

#### Urine Pharmacokinetic Results

Comparing LS mean ratios, cumulative excretion of cyclobenzaprine was approximately 11%, 15%, and 21% lower for Formulations A, B, and C, respectively, compared with oral IR cyclobenzaprine HCl. For Formulation A, cumulative excretion of norcyclobenzaprine and cyclobenzaprine‐N‐glucuronide was approximately 64% and 57%, respectively, of oral IR cyclobenzaprine HCl, and cumulative excretion of the metabolites for Formulations B and C was approximately 72%‐75% and 91%‐114% of that observed following administration of oral IR cyclobenzaprine HCl.

#### Safety

Across the four treatment groups, a total of 16 AEs were reported by 13 of the 24 participants (Table 3): 3 participants (50%) receiving Formulation A, 5 (83%) receiving Formulation B, 5 (83%) receiving Formulation C, and 0 receiving oral IR cyclobenzaprine HCl. There were no serious AEs or discontinuations due to AEs in this study. All reported AEs were considered mild. The only AEs reported by ≥2 participants in any treatment group were oral hypoesthesia (Formulations A, B, and C and oral IR cyclobenzaprine HCl: n = 2, 3, 3, 0) and somnolence (n = 0, 2, 1, 0). There were no clinically meaningful changes in clinical laboratory parameters, vital signs, or ECG in any treatment group.

**Table 3:** Study 1: Summary of Safety (Safety Population)

	Formulation A:	Formulation B:	Formulation C:	
Participants with AE, n (%)	Sublingual Cyclobenzaprine HCl 2.8 mg (Potassium Phosphate Dibasic) (n = 6)	Sublingual Cyclobenzaprine HCl 2.8 mg (Sodium Phosphate Dibasic) (n = 6)	Sublingual Cyclobenzaprine HCl 2.8 mg (Trisodium Citrate) (n = 6)	Oral Cyclobenzaprine HCl 5 mg IR (n = 6)
≥1 AE	3 (50)	5 (83)	5 (83)	0
≥1 serious AE	0	0	0	0
All AEs				
Oral hypoesthesia	2 (33)	3 (50)	3 (50)	0
Somnolence	0	2 (33)	1 (17)	0
Oral mucosal erythema	1 (17)	0	1 (17)	0
Nausea	0	1 (17)	0	0
Oral discomfort	0	1 (17)	0	0
Hot flash	0	1 (17)	0	0

AE, adverse event; IR, immediate release.

### Study 2

#### Participants

Between October 14, 2019, and January 10, 2020, a total of 22 of 38 screened healthy volunteers were enrolled, and 16 participants received ≥1 dose of study medications, with 15 participants completing all treatment periods. The mean age was 52.7 years, and there was an equal proportion of male to female participants (Table 4).

**Table 4:** Study 2: Demographic and Baseline Characteristics (Safety Population)

Characteristic	Overall (N = 16)
Mean age (range), years	52.7 (36, 62)
Sex, n (%)	
Female	8 (50)
Male	8 (50)
Race, n (%)	
White	16 (100)
Ethnicity, n (%)	
Not Hispanic	14 (88)
Hispanic	2 (12)
Mean BMI (range), kg/m^2^	25.9 (21.2, 28.8)

BMI, body mass index.

#### Dose‐Proportionality and Food Effect

**Figure 2:**
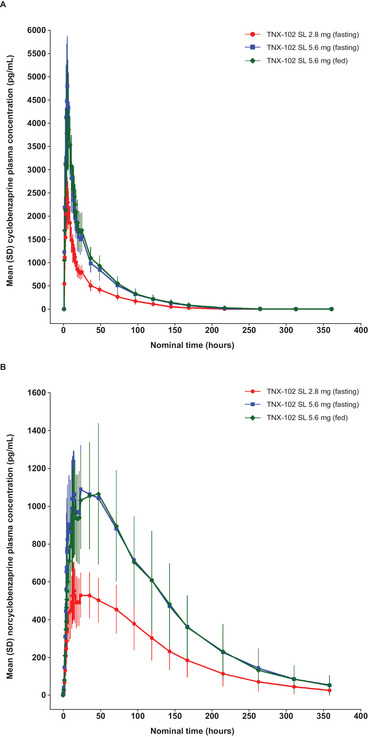
Study 2: Plasma concentration of (A) cyclobenzaprine and (B) norcyclobenzaprine for TNX‐102 SL 2.8 mg and TNX‐102 SL 5.6 mg under fasting and fed states (pharmacokinetic population). SL, sublingual.

The PK parameters for cyclobenzaprine and norcyclobenzaprine under all three conditions were adequately characterized (Table 5). Plasma concentration–time profiles under fasting conditions showed rapid absorption with a median time to T_max_ for cyclobenzaprine of 4.3 h and for norcyclobenzaprine of 15.0 h for sublingual cyclobenzaprine HCl 2.8 mg and of 4.3 and 35.6 h, respectively, for sublingual cyclobenzaprine HCl 5.6 mg (Figure 2). For cyclobenzaprine, no statistically significant differences were found between treatments, periods, or sequences for dose‐normalized (to 2.8 mg) ln‐transformed AUC_0‐t_, AUC_0‐∞_, or C_max_, and no statistically significant differences between treatments were found for untransformed T_max_, K_el_, and T_1/2_ (Table S2). The 90% CIs for the ratio of geometric means of the ln‐transformed dose‐normalized AUC_0‐t_, AUC_0‐∞_, and C_max_ were within the acceptance range of 80.0%‐125.0%, meeting criteria for dose‐proportionality.

**Table 5:** Study 2: Single‐Dose Pharmacokinetic Parameters for Cyclobenzaprine and Norcyclobenzaprine in Plasma (Pharmacokinetic Population)

Parameter	TNX‐102 SL 2.8 mg (Fasting) (n = 15)	TNX‐102 SL 5.6 mg (Fasting) (n = 16)	TNX‐102 SL 5.6 mg (Fed) (n = 16)
Cyclobenzaprine			
AUC_0‐t_, mean (SD), ng·h/mL	64.4 (14.1)	128.1 (27.6)	133.1 (27.6)
AUC_0‐∞_, mean (SD), ng·h/mL	67.7 (14.2)	132.3 (28.1)	136.9 (27.7)
Residual area, %	5.2 (1.6)	3.2 (0.9)	2.8 (0.7)
C_max_, mean (SD), ng/mL	2.5 (0.4)	5.1 (1.0)	4.5 (1.0)
T_max_, median (range), h	4.3 (1.0, 5.7)	4.3 (1.0, 5.7)	4.7 (4.0, 10.0)
K_el_, mean (SD), L/h	0.020 (0.004)	0.020 (0.003)	0.019 (0.004)
t_1/2_, mean (SD), h	35.4 (7.2)	36.4 (7.6)	37.9 (7.1)
Norcyclobenzaprine			
AUC_0‐t_, mean (SD), ng·h/mL	79.4 (26.7)	158.1 (50.3)	156.2 (56.2)
AUC_0‐∞_, mean (SD), ng·h/mL	82.4 (30.1)	164.1 (55.8)	161.8 (61.1)
Residual area, %	2.9 (2.6)	3.0 (2.7)	2.9 (2.5)
C_max_, mean (SD), ng/mL	0.6 (0.1)	1.2 (0.2)	1.1 (0.4)
T_max_, median (range), h	15.0 (13.0, 48.0)	35.6 (6.0, 72.1)	35.8 (13.0, 48.9)
K_el_, mean (SD), L/h	0.012 (0.003)	0.012 (0.003)	0.012 (0.003)
t_1/2_, mean (SD), h	61.3 (17.3)	63.0 (16.9)	62.0 (16.3)

AUC, area under the plasma concentration‐versus‐time curve; AUC_0‐t_, AUC from time 0 to last measurable concentration; AUC_0‐∞_, AUC from time 0 extrapolated to infinity; C_max_, maximum measured plasma concentration; K_el_, elimination constant; SL, sublingual; t_1/2_, elimination half‐life; T_max_, time of the maximum measured plasma concentration.

#### Cyclobenzaprine and Norcyclobenzaprine Pharmacokinetics in Plasma

Plasma concentration–time profiles for cyclobenzaprine and norcyclobenzaprine under fed and fasting conditions were generally similar, showing rapid absorption and comparable AUC_0‐t_, AUC_0‐∞_, C_max_, and T_max_ (Figure 2 and Table 5). For cyclobenzaprine, no statistically significant differences were found between treatments, periods, or sequences in ln‐transformed AUC_0‐t_ and AUC_0‐∞_ and in untransformed T_max_, K_el_, and t_1/2_ (Table S3). A statistically significant difference was found between treatments for C_max_; however, the 90% CIs for the ratio of geometric means of the ln‐transformed AUC_0‐t_, AUC_0‐∞_, and C_max_ were within the acceptance range of 80.0%‐125.0%, meeting criteria for lack of food effect. Notably, in Study 2, the larger sample size and longer duration compared with Study 1 allow for a more accurate reporting of the t_1/2_ for norcyclobenzaprine, which was 61.3 and 63.0 h for 2.8 and 5.6 mg, respectively.

#### Safety

Across the three periods, a total of 61 AEs were reported by 14 of the 16 participants (Table 6). Most reported AEs (53 of 61 [87%]) were mild in severity. The most commonly reported AEs were somnolence (44% of participants overall), oral hypoesthesia (38%), nasopharyngitis (31%), abnormal product taste (31%), and headache (19%). AEs related to oral hypoesthesia were mild and considered probably related to the study drug. One participant withdrew from the study after Period 2 as a precautionary measure owing to TEAEs of “hyperesthesia teeth” and “mouth ulceration.” Overall, across all periods, two participants experienced abnormalities related to vital signs (blood pressure increase); each report was considered mild in severity and possibly related to treatment and resolved spontaneously. There were no other clinically meaningful changes in clinical laboratory parameters, vital signs, or ECG in any treatment group.

**Table 6:** Study 2: Summary of Safety (Safety Population)

Participants With AE, n (%)	TNX‐102 SL 2.8 mg (Fasting) (n = 15^a^)	TNX‐102 SL 5.6 mg (Fasting) (n = 16)	TNX‐102 SL 5.6 mg (Fed) (n = 16)
≥1 AE	5 (33)	10 (63)	10 (63)
≥1 Serious AE	0	0	0
Discontinuations due to AE	0	0	1 (7)
AEs occurring in ≥2 participants in any treatment group			
Oral hypoesthesia	2 (13)	3 (19)	4 (25)
Somnolence	1 (7)	4 (25)	3 (19)
Product tastes abnormal	0	3 (19)	3 (19)
Nasopharyngitis	3 (20)	1 (6)	1 (6)
Headache	2 (13)	1 (6)	1 (6)

AE, adverse event; SL, sublingual.

a One patient discontinued before completing the treatment period of TNX‐102 SL 2.8 mg in a fasted state.

## Discussion

Daily oral cyclobenzaprine HCl tablets have shown transient benefits in FM, which is a chronic pain condition. To improve on this treatment effect, we explored whether certain basic excipients may drive transmucosal absorption of cyclobenzaprine with sublingual administration. The two studies reported here were conducted to assess the pharmacokinetics of different sublingual cyclobenzaprine HCl formulations in order to identify a formulation for further development and then to evaluate the dose proportionality and food effect of the selected formulation.

The findings of Study 1 indicated that sublingual cyclobenzaprine HCl 2.8 mg formulated with K_2_HPO_4_ was associated with greater early absorption (AUC_0‐1_ and AUC_0‐2_) compared with sublingual cyclobenzaprine HCl 2.8 mg formulated with Na_2_HPO_4_ or Na_3_C_6_H_5_O_7_. The AUC_0‐1_ and AUC_0‐2_ for sublingual cyclobenzaprine HCl formulated with K_2_HPO_4_ were 4.4‐fold and ∼2‐fold higher, respectively, than for oral cyclobenzaprine HCl IR, whereas the mean AUC_8‐24_ was ∼22% lower. When doses were normalized, mean AUC_0‐1_, AUC_0‐2_, and AUC_8‐24_ for sublingual cyclobenzaprine HCl formulated with K_2_HPO_4_ (Formulation A) were 7.8‐fold, 3.3‐fold, and 1.4‐fold higher, respectively, than for oral cyclobenzaprine HCl IR. The absorption lag time was substantially shorter, with a ∼10‐fold difference in all three formulations compared with oral cyclobenzaprine HCl IR. These findings suggest a faster onset of absorption after sublingual administration compared with oral cyclobenzaprine administration. Furthermore, the rate of absorption for sublingual cyclobenzaprine HCl 2.8 mg formulated with K_2_HPO_4_ was substantially shorter than that for oral cyclobenzaprine HCl 5 mg IR, suggesting that the absorption of cyclobenzaprine into the systemic circulation starts much earlier after administration of sublingual cyclobenzaprine HCl than oral cyclobenzaprine HCl 5 mg IR. The overall PK profile over the remainder of the 48‐h post‐dose sampling interval was generally similar for the sublingual cyclobenzaprine HCl 2.8 mg formulations and oral cyclobenzaprine HCl 5 mg IR, suggesting that there was little appreciable local accumulation of sublingual cyclobenzaprine HCl within the oral mucosa. In addition, the mean bioavailability of cyclobenzaprine HCl in plasma for sublingual cyclobenzaprine HCl formulated with K_2_HPO_4_ relative to oral cyclobenzaprine HCl 5 mg IR was approximately 154%. Based on these results, the tablet formulated with K_2_HPO_4_ was selected for further clinical development and designated TNX‐102 SL. In Study 2, sublingual TNX‐102 SL showed dose‐proportionality and lack of food effect.

Sublingual administration of TNX‐102 SL provided a more rapid absorption compared with oral, swallowed administration, with peak exposure of cyclobenzaprine in the blood following bedtime administration aligned with the sleeping period to minimize morning somnolence. The rapid absorption of cyclobenzaprine from sublingual TNX‐102 SL relative to oral cyclobenzaprine HCl 5 mg IR provides a greater diurnal peak‐to‐trough variation in plasma cyclobenzaprine and is expected to provide greater therapeutic benefit by increasing exposure during sleep and reducing daytime exposure. Additional pharmacodynamic effects of cyclobenzaprine HCl treatment that may be beneficial for people with FM include countering the arousal effects of an activated noradrenergic system,^17^ improving sleep disturbance,^18^ and modulating sleep architecture.^19^

The 48‐h sampling time period of Study 1 comparing sublingual cyclobenzaprine HCl formulations to oral cyclobenzaprine HCl was too short to accurately assess PK parameters for norcyclobenzaprine, as confirmed by the long terminal half‐life of norcyclobenzaprine, which was more than 60 h in Study 2 that had a 360‐h sampling period (Table 5).

Bypassing first‐pass hepatic metabolism via transmucosal absorption, resulting in lower exposure to norcyclobenzaprine, is expected to contribute to apparent reductions in daytime side effects as observed in a phase 3 trial of sublingual cyclobenzaprine HCl formulated with K_2_HPO_4_^15^ compared with oral cyclobenzaprine HCl IR.^7^ A pivotal multi‐dose bridging PK and safety study showed that cyclobenzaprine steady‐state levels and AUC were higher than norcyclobenzaprine during the first 8 h after sublingual TNX‐102SL administration, optimizing the effects of cyclobenzaprine on the sleeping brain.^14^ For patients receiving oral cyclobenzaprine HCl IR, accumulation of norcyclobenzaprine, which generally antagonizes the same receptors as cyclobenzaprine HCl with reduced potency,^14^, ^20^, ^21^ may limit the durability of efficacy in FM, and reduced norcyclobenzaprine accumulation could improve the durability of effect for patients receiving sublingual cyclobenzaprine HCl. Furthermore, norcyclobenzaprine binds more avidly to the norepinephrine transporter and has an active inhibitory effect on the transporter, potentially interfering with sleep.^14^

Urinary excretion of cyclobenzaprine and norcyclobenzaprine showed that the cumulative amount excreted for cyclobenzaprine was slightly lower for sublingual cyclobenzaprine HCl 2.8 mg with K_2_HPO_4_ compared with oral cyclobenzaprine HCl 5 mg IR, whereas there was a more pronounced decrease in norcyclobenzaprine and cyclobenzaprine‐N‐glucuronide metabolites for this formulation (approximately 64% and 57% of that following administration of the cyclobenzaprine HCl 5 mg IR tablet).

Predictable and consistent dosing is necessary to ensure sufficient drug exposure that is associated with an appropriate clinical response and limited potential for adverse effects and toxicity. After a single administration in healthy volunteers, sublingual cyclobenzaprine HCl formulated with K_2_HPO_4_ showed dose proportionality between the 2.8 and 5.6 mg doses. Additionally, sublingual cyclobenzaprine HCl 5.6 mg was rapidly absorbed in both a fasted and fed state and showed similar PK parameters, suggesting that absorption, metabolism, and elimination of sublingual cyclobenzaprine HCl are similar regardless of meals. Taken together, results suggest that sublingual TNX‐102 SL provides reliable PK properties and warrants further investigation as a potential daily bedtime treatment for FM.

Single‐dose administration of sublingual cyclobenzaprine HCl formulated with K_2_HPO_4_ was generally well tolerated by healthy adult volunteers. Across both studies, all reported TEAEs were mild or moderate in severity. Transient oral hypoesthesia and somnolence were the most frequently reported AEs. It is important to note that in contrast to morning dosing in these two PK studies, the dosing regimen in the Phase 3 studies of sublingual cyclobenzaprine HCl is nightly at bedtime, at which time somnolence is a positive attribute during the sleep period.^15^, ^16^ Oral hypoesthesia/paresthesia following treatment with sublingual cyclobenzaprine HCl may relate to potential local anesthetic properties of cyclobenzaprine, a tricyclic molecule that differs from the tricyclic antidepressant amitriptyline by a single double bond in the cycloheptane ring. Tricyclic antidepressants such as amitriptyline have local anesthetic properties as a result of the potent neuronal sodium channel–blocking activity.^22^, ^23^, ^24^ Previous reports have shown that a droplet of the liquid formulation of amitriptyline (∼100 mmol/L) on the tongue results in local numbness lasting about 1 h.^25^ Similarly, cyclobenzaprine was found to inhibit voltage‐gated sodium channels Nav1.7 and Nav1.8 by 99.9% and 95.8%, respectively, at 30 µM in vitro on cloned human ion channels (unpublished observations). Thus, the local numbing effect observed clinically with sublingual cyclobenzaprine HCl is presumed to be an expected tricyclic molecular effect resulting from sodium channel blockade on regional sensory neurons at the site of absorption. Results of the other safety evaluations in these single‐dose studies further support the tolerability of sublingual cyclobenzaprine HCl, with no clinically meaningful changes in clinical laboratory tests, vital sign measurements, or ECG parameters observed in any treatment group or condition.

Limitations of these studies include the small sample size and the inclusion of healthy volunteers rather than participants with a disease. However, PK studies early in the process of drug development are typically small and include healthy populations. While noncompartmental analysis was used in both studies for direct estimation of PK parameters as reported in Tables 2 and 5, a model‐based PK analysis was also performed in Study 1 as a secondary analysis method to obtain absorption‐related parameters, particularly the absorption lag time, for differentiation between the candidate formulations. Although model‐based analyses may introduce bias due to their assumptions on the underlying model structure and thus may be considered less reliable than noncompartmental analyses,^26^ this approach provided valuable additional information in selecting the formulation to carry forward into Study 2. The fed state used in Study 2 may not be representative of an individual's typical meal, and limits the ability to draw conclusions on the effects of all foods.

## Conclusions

Two Phase 1 studies were conducted to select and characterize a sublingual tablet containing cyclobenzaprine HCl with transmucosal absorption for further clinical development. In Study 1, single‐dose administration of sublingual cyclobenzaprine HCl 2.8 mg formulated with three different basifying agents (K_2_HPO_4_, Na_2_HPO_4_, and Na_3_C_6_H_5_O_7_) showed predictable PK parameters with faster absorption, higher peak plasma concentrations when dose normalized, and increased bioavailability relative to oral, swallowed cyclobenzaprine HCl and was well tolerated in healthy adult volunteers. Each of the basifying agents conferred transmucosal absorption based on the rapid absorption rate. Based on the pharmacokinetics, sublingual cyclobenzaprine HCl 2.8 mg formulated with K_2_HPO_4_ as the basifying agent was selected for further clinical development. TNX‐102 SL is a small, rapidly disintegrating tablet containing 2.8 mg of cyclobenzaprine HCl formulated with K_2_HPO_4_ in a eutectic formulation and is designed for sublingual administration and transmucosal absorption. In Study 2, the PK parameters of sublingual cyclobenzaprine HCl demonstrated dose proportionality between 2.8 and 5.6 mg and were not affected by a high‐calorie, high‐fat meal. Taken together, these data confirm that the design objectives of the sublingual cyclobenzaprine HCl tablet were achieved in terms of transmucosal absorption and bypassing first‐pass hepatic metabolism. Subsequently, in two Phase 3 multicenter randomized, double‐blind, placebo‐controlled studies in patients with FM, treatment with TNX‐102 SL (sublingual cyclobenzaprine HCl formulated with K_2_HPO_4_) 5.6 mg (2 × 2.8 mg tablets) at bedtime resulted in significant reductions in the prespecified primary endpoint of daily pain intensity and was well tolerated.^15^, ^16^ These data suggest that the novel, sublingual formulation is associated with improved efficacy and tolerability relative to oral cyclobenzaprine HCl in chronic dosing for FM. In August 2025, TNX‐102 SL was approved by the FDA for the treatment of FM as a bedtime therapy.^3^

## Conflicts of Interest

BLD, GMS, and SL are employees of Tonix Pharmaceuticals, Inc. (Berkeley Heights, NJ) and own stock and/or have options in the company. BM is a paid consultant for Tonix Pharmaceuticals. EG is an employee of Tonix Medicines and owns stock and/or has options in Tonix Pharmaceuticals Holding Corp. This study and medical writing support for preparation of this manuscript were funded by Tonix Pharmaceuticals, Inc. Tonix Pharmaceuticals, Inc., was involved in the study design; in the collection, analysis, and interpretation of data; in the writing of the report; and in the decision to submit the article for publication.

## Funding

This study and medical writing support for the preparation of this manuscript were funded by Tonix Pharmaceuticals, Inc. (Berkeley Heights, New Jersey). Tonix Pharmaceuticals, Inc., was involved in the study design; in the collection, analysis, and interpretation of data; in the writing of the report; and in the decision to submit the article for publication.

## Supporting information



Supplemental Information
Additional supplemental information can be found by clicking the Supplements link in the PDF toolbar or the Supplemental Information section at the end of the web‐based version of this article.

## Data Availability

Deidentified study data will be made available upon reasonable request. Access to deidentified study data will be provided upon application, and all requests will be reviewed by the trial sponsor due to the limited data from a small sample size of patients. Data will be shared under agreements designed to protect against participant reidentification, and data will be provided in a secure research environment, further protecting participant privacy.
