# Supplementary material for: Single‐Dose Pharmacokinetic Assessment of TNX‐102 SL (Cyclobenzaprine HCl Sublingual Tablets): Results From Randomized, Open‐Label Studies in Healthy Volunteers
Source: Clin Pharmacol Drug Dev. 2026 Feb 26;15(3):e70034. doi: 10.1002/cpdd.70034 (PMC12946586; doi:10.1002/cpdd.70034)
Supplement: Supplementary file 1 — Supplemental Information Additional supplemental information can be found by clicking the Supplements link in the PDF toolbar or the Supplemental Information section at the end of the web‐based version of this article. [file CPDD-15-0-s001.docx]

Supplementary Table 1. Study 1: Single-Dose Cyclobenzaprine Exposure in Plasma

| Mean (SD) | Formulation A:  sublingual cyclobenzaprine HCl 2.8 mg (potassium phosphate dibasic)  (n=6) | Formulation B:  sublingual cyclobenzaprine HCl 2.8 mg (sodium phosphate dibasic)  (n=6) | Formulation C:  sublingual cyclobenzaprine HCl 2.8 mg (trisodium citrate)  (n=6) | Oral cyclobenzaprine HCl 5 mg IR  (n=6) | Ratio  (Formulation A/ Oral IR cyclobenzaprine HCl) |
| --- | --- | --- | --- | --- | --- |
| Cyclobenzaprine |  |  |  |  |  |
| AUC_0-t_ ng*h/mL | 57.4 (10.7) | 49.0 (10.4) | 46.8 (9.2) | 69.5 (18.8) | - |
| Dose normalized | 20.5 | 17.5 | 16.7 | 13.9 | 1.5 |
| AUC_0-∞_ ng*h/mL | 78.8 (15.9) | 64.4 (15.6) | 64.1 (14.5) | 91.4 (24.2) | - |
| Dose normalized | 28.1 | 23.0 | 22.9 | 18.3 | 1.5 |
| AUC_0-1_ ng*h/mL | 0.88 (0.53)^a^ | 0.61 (0.56)^b^ | 0.68 (0.16)^a^ | 0.20 (0.24) | - |
| Dose normalized | 0.31 | 0.22 | 0.24 | 0.04 | 7.8 |
| AUC_0-2_ ng*h/mL | 2.8 (1.4)^b^ | 2.1 (1.2) | 2.1 (0.4) | 1.5 (1.2) | - |
| Dose normalized | 1.0 | 0.74 | 0.76 | 0.31 | 3.3 |
| AUC_0-8_ ng*h/mL | 19.3 (5.6) | 15.3 (3.2)^b^ | 14.8 (2.9)^b^ | 21.3 (5.7) | - |
| Dose normalized | 6.9 | 5.5 | 5.3 | 4.3 | 1.6 |
| AUC_8-24_, ng*h/mL | 21.5 (4.3) | 19.2 (4.4)^b^ | 17.6 (3.2)^a^ | 27.7 (8.6) | - |
| Dose normalized | 7.7 | 6.9 | 6.3 | 5.5 | 1.4 |
| AUC_0-24_ ng*h/mL | 40.7 (8.3) | 34.6 (6.6)^b^ | 32.4 (5.5)^a^ | 49.1 (13.8) | - |
| Dose normalized | 14.6 | 12.3 | 11.6 | 9.8 | 1.5 |
| C_max_ ng/mL | 3.4 (1.0) | 2.7 (0.5) | 2.9 (0.9) | 4.3 (1.4) | - |
| Dose normalized | 1.2 | 0.98 | 1.0 | 0.85 | 1.4 |

AUC, area under the plasma concentration-versus-time curve; AUC_0-1_, AUC from time 0 to 1 hour post-dose; AUC_0‑2_, AUC from time 0 to 2 hours post-dose; AUC_0-8_, AUC from time 0 to 8 hours post-dose; AUC_8-24_, AUC from time 8 to 24 hours post-dose; AUC_0‑48_, AUC from time 0 to 48 hours post-dose; IR, immediate release. ^a^Nominal *P*<0.01 vs Oral IR cyclobenzaprine HCl. ^b^Nominal *P*<0.05 vs Oral IR cyclobenzaprine HCl.

Supplementary Table 2. Study 2: Statistical Analysis of Cyclobenzaprine and Norcyclobenzaprine Pharmacokinetic Parameters for the Comparison of TNX-102 SL 2.8 and 5.6 mg (Dose Normalized*)

| Parameter, geometric LS mean | TNX-102 SL 2.8 mg (fasting)  (n=15) | TNX-102 SL 5.6 mg^a^ (fasting)  (n=16) | Ratio (%) | 90% Geometric CI | Sequence  *P* value | Period  *P* value | Treatment  *P* value |
| --- | --- | --- | --- | --- | --- | --- | --- |
| Cyclobenzaprine | | | | | | | |
| AUC_0-t_, ng*h/mL | 63.864 | 63.739 | 100.2 | 94.5, 106.2 | 0.3826 | 0.2524 | 0.9528 |
| AUC_0-∞_, ng*h/mL | 67.328 | 65.834 | 102.3 | 96.8, 108.0 | 0.3674 | 0.2693 | 0.4774 |
| C_max_, ng/mL | 2.474 | 2.528 | 97.9 | 92.1, 104.1 | 0.2843 | 0.7102 | 0.5462 |
| Norcyclobenzaprine | | | | | | | |
| AUC_0-t_, ng*h/mL | 75.007 | 75.666 | 99.1 | 92.2, 106.6 | 0.2864 | 0.9032 | 0.8329 |
| AUC_0-∞_, ng*h/mL | 77.315 | 78.101 | 99.0 | 91.8, 106.8 | 0.3515 | 0.9099 | 0.8152 |
| C_max_, ng/mL | 0.557 | 0.572 | 97.5 | 91.3, 104.0 | 0.0034 | 0.1709 | 0.4921 |

AUC, area under the plasma concentration-versus-time curve; AUC_0-t_, AUC from time 0 to last measurable concentration; AUC_0‑∞_, AUC from time 0 extrapolated to infinity; C_max_, maximum measured plasma concentration; LS, least squares; SL, sublingual. ^a^Dose normalized to 2.8 mg.

Supplementary Table 3. Study 2: Statistical Analysis of Cyclobenzaprine and Norcyclobenzaprine Pharmacokinetic Parameters for the Comparison of TNX-102 SL 5.6 mg in the Fasted and Fed State

| Parameter, geometric LS mean | TNX-102 SL 5.6 mg (fasting)  (n=15) | TNX-102 SL 5.6 mg (fed)  (n=6) | Ratio (%) | 90% Geometric CI | Sequence  *P* value | Period  *P* value | Treatment  *P* value |
| --- | --- | --- | --- | --- | --- | --- | --- |
| Cyclobenzaprine | | | | | | | |
| AUC_0-t_, ng*h/mL | 126.373 | 131.497 | 104.1 | 99.0, 109.3 | 0.4623 | 0.1597 | 0.1792 |
| AUC_0-∞_, ng*h/mL | 130.502 | 135.294 | 103.7 | 98.6, 109.0 | 0.4578 | 0.1965 | 0.2265 |
| C_max_, ng/mL | 4.994 | 4.446 | 89.0 | 82.1, 96.6 | 0.5954 | 0.2154 | 0.0254 |
| Norcyclobenzaprine | | | | | | | |
| AUC_0-t_, ng*h/mL | 152.182 | 146.254 | 96.1 | 89.5, 103.2 | 0.3394 | 0.1330 | 0.3378 |
| AUC_0-∞_, ng*h/mL | 157.116 | 150.695 | 95.9 | 89.2, 103.2 | 0.3914 | 0.1295 | 0.3292 |
| C_max_, ng/mL | 1.151 | 1.093 | 94.9 | 87.7, 102.7 | 0.0150 | 0.7247 | 0.2625 |

AUC, area under the plasma concentration-versus-time curve; AUC_0-t_, AUC from time 0 to last measurable concentration; AUC_0‑∞_, AUC from time 0 extrapolated to infinity; C_max_, maximum measured plasma concentration; LS, least squares; SL, sublingual.
